# Associations between fatty acid intake and diabetic retinopathy in a Japanese population

**DOI:** 10.1038/s41598-023-39734-x

**Published:** 2023-08-09

**Authors:** Mariko Sasaki, Kenya Yuki, Akiko Hanyuda, Kazumasa Yamagishi, Kaoru Motomura, Toshihide Kurihara, Yohei Tomita, Kiwako Mori, Nobuhiro Ozawa, Yoko Ozawa, Norie Sawada, Kazuno Negishi, Kazuo Tsubota, Shoichiro Tsugane, Hiroyasu Iso

**Affiliations:** 1 Department of Ophthalmology, Keio University School of Medicine, 35 Shinanomachi, Shinjuku-ku, Tokyo, 160-8582 Japan; 2 National Hospital Organization Tokyo Medical Center, 2-5-1 Higashigaoka Meguro-ku, Tokyo, 152-8902 Japan; 3 Department of Public Health Medicine, Faculty of Medicine, and Health Services Research and Development Center, University of Tsukuba, 1-1-1 Tennodai, Tsukuba, Ibaraki 305-8575 Japan; 4 Ibaraki Western Medical Center, 555 Otsuka, Chikusei, Ibaraki 308-0813 Japan; 5 St.Luke’s International Hospital, 9-1 Akashi-cho, Chuo-ku, Tokyo, 104-8560 Japan; 6 Divison of Cohort Research, National Cancer Center Institute for Cancer Control, 5-1-1 Tsukiji, Chuo-ku, Tokyo, 104-0045 Japan; 7 National Institute of Health and Nutrition, National Institutes of Biomedical Innovation, Health and Nutrition, 1-23-1 Toyama, Shinjuku-ku, Tokyo, 162-8636 Japan; 8 Institute for Global Health Policy Research, Bureau of International Health Cooperation, National Center for Global Health and Medicine, 1-21-1 Toyama, Shinjuku-ku, Tokyo, 162-8655 Japan

**Keywords:** Health care, Medical research, Risk factors

## Abstract

Residents of Chikusei City, aged 40–74 years, underwent systemic and ophthalmological screening, and participants with diabetes were included in this analysis. Dietary intake was assessed using a food frequency questionnaire and calculated as a percentage of the total energy. The presence of diabetic retinopathy (DR) was defined as Early Treatment Diabetic Retinopathy Study levels ≥ 20 in either eye. The association between dietary fatty acid intake and DR has been examined in a cross-sectional study. Among the 647 diabetic participants, 100 had DR. The mean total fat and saturated fatty acid (SFA) intakes were 22.0% and 7.3% of the total energy intake, respectively. After adjusting for potential confounders, the highest quartiles of total fat and SFA intake were positively associated with the presence of DR compared with the lowest quartiles (odds ratios (95% confidence intervals), 2.61 (1.07–6.39), p for trend = 0.025, and 2.40 (1.12–5.17), p for trend = 0.013, respectively). No significant associations were found between DR prevalence and monounsaturated or unsaturated fatty acid intake. These results suggest that a high intake of fat and SFA may affect the development of DR, even in individuals whose total fat intake is generally much lower than that of Westerners.

## Introduction

Diabetic retinopathy (DR) is the leading cause of visual impairment in working adults^1^, and is also the most common microvascular complication of diabetes. According to a meta-analysis of 59 population-based studies, the global prevalences of DR and vision-threatening DR were 22.27% and 6.17%, respectively, with an estimated 103.12 million individuals living with DR and 28.54 million living with vision-threatening DR in 2020^2^. The major risk factors for DR include prolonged duration of diabetes and poor control of glycemia, blood pressure, and lipids^1,3^.

Nutrition plays an important role in the pathogenesis and prevention of ocular diseases. Dietary intake of n-3 polyunsaturated fatty acids (PUFA) by fish is associated with a lower risk of age-related macular degeneration (AMD)^4–7^. A meta-analysis suggested that n-3 PUFA intake reduces the risk of late AMD, whereas fish consumption reduces the risk of both late and early AMD^8^. Meanwhile, we recently reported that a higher intake of saturated fatty acid (SFA) in a population with low mean SFA intake was associated with a lower risk of early AMD^9^, suggesting that the association between fatty acid intake and AMD could differ among populations with different genetic backgrounds or dietary patterns.

Although the association between fatty acid intake and the risk of developing diabetes has not been fully elucidated, several studies have reported that SFA intake is a risk factor for developing diabetes, whereas PUFA intake may reduce this risk^10–13^. In addition, growing evidence suggests that PUFA intake has a beneficial effect on complications associated with diabetes. Omega-3 PUFA supplementation favorably modified cardiometabolic biomarkers, lipids, glycemia, and pro-inflammatory cytokines in type 2 diabetes^14^. A randomized controlled trial showed that the administration of n-3 PUFA supplements attenuated the progression of albuminuria in individuals with type 2 diabetes mellitus and a history of coronary artery disease^15^. The National Health and Nutrition Examination Survey in the United States reported that dietary intake of n-6 PUFA and linolenic acid is associated with a lower risk of peripheral neuropathy^16^. Asian populations consume smaller amounts of SFA-containing foods than Western populations^17,18^, and a meta-analysis reported ethnic differences in the relationship between insulin sensitivity and response^19^. However, studies examining the association between fatty acid intake and DR have shown inconsistent results^20–24^, and such studies have not been conducted among Asians.

Since dyslipidemia is a potential risk factor for DR^25–27^, dietary intake of fatty acids may affect lipid metabolism and the pathogenesis of DR. Therefore, we aimed to examine the cross-sectional association between dietary fatty acid intake and the prevalence of DR in participants with diabetes in a Japanese population-based cohort, the Japan Public Health Center-based Prospective Study for the Next Generation (JPHC-NEXT) Eye Study.

## Results

In total, 647 and 100 participants were identified as having diabetes and DR, respectively. The characteristics of the 647 patients with DM are presented in Table 1. The mean total energy intake was 2292.5 kilocalories (kcal), and the total fat and SFA intakes were 22.0% and 7.3% of the total energy intake (% energy), respectively. There was no significant difference in total energy intake between patients with and without DR. The intakes of total fat, SFA, PUFA, and n6-PUFA were significantly higher in patients with DR than in those without DR. Conversely, there was no significant difference in the intake of MUFA and n3-PUFA. The patients with and without DR were similar in terms of age, sex, body mass index (BMI), smoking status, hypertension, total cholesterol (TC), high-density lipoprotein cholesterol (HDL-C), low-density lipoprotein cholesterol (LDL-C), and intake of α-tocopherol, β-carotene, vitamin C and vitamin D. Patients with DR had higher glycated hemoglobin (HbA1c) levels and protein intake, lower serum triglyceride (TG) levels, less dyslipidemia, and were less likely to be current drinkers.

**Table 1:** Baseline characteristics stratified by the presence of diabetic retinopathy.

Variables	All subjects	No DR	DR	*P* for difference
	No. (%) or mean (standard deviation)			
N	647	547	100	
Age, years	65.2 (7.0)	65.3 (6.9)	64.8 (7.3)	0.552
Sex, % male	368 (56.9)	307 (56.1)	61 (61.0)	0.365
BMI, kg/m^2^	24.8 (3.8)	24.8 (3.8)	24.8 (3.9)	0.972
SBP, mmHg	132.6 (17.0)	132.0 (16.7)	135.8 (18.3)	0.080
DBP, mmHg	76.0 (11.0)	75.9 (11.0)	76.4 (11.4)	0.809
Smoking status, % current	101 (15.6)	83 (15.2)	18 (18.0)	0.474
Alcohol, % current	198 (30.6)	177 (32.4)	21 (21.0)	**0.023**
Hypertension, %	427 (66.0)	357 (65.3)	70 (70.0)	0.358
Dyslipidemia, %	451 (69.7)	392 (71.7)	59 (59.0)	**0.011**
HbA1c, %,	7.0 (1.2)	6.9 (1.2)	7.5 (1.2)	** < 0.0001**
Total cholesterol, mmol/L	5.15 (0.94)	5.17 (0.94)	5.04 (0.96)	0.188
HDL- cholesterol, mmol/L	1.50 (0.38)	1.49 (0.38)	1.53 (0.39)	0.469
LDL- cholesterol, mmol/L	3.12 (0.84)	3.13 (0.84)	3.01 (0.83)	0.187
Triglycerides, mmol/L	1.48 (0.91)	1.52 (0.93)	1.26 (0.79)	**0.011**
Creatinine, mg/dL	0.76 (0.25)	0.76 (0.24)	0.77 (0.28)	0.809
Total calorie, kcals	2292.5 (1359.3)	2282.1 (1399.8)	2349.8 (1115.9)	0.594
Total fat, % energy	22.01 (6.90)	21.75 (6.92)	23.40 (6.64)	**0.028**
SFA, % energy	7.28 (2.76)	7.19 (2.77)	7.78 (2.62)	**0.048**
MUFA, % energy	9.21 (3.33)	9.11 (3.32)	9.77 (3.33)	0.068
PUFA, % energy	5.50 (1.68)	5.44 (1.66)	5.82 (1.73)	**0.036**
n3-PUFA, % energy	1.02 (0.45)	1.01 (0.44)	1.07 (0.47)	0.165
n6-PUFA, % energy	4.47 (1.36)	4.42 (1.35)	4.73 (1.40)	**0.034**
Protein, % energy	14.20 (2.98)	14.09 (2.97)	14.81 (2.97)	**0.042**
α-Tocofenol, mg/1000 kcal	3.71 (1.43)	3.70 (1.45)	3.76 (1.26)	0.781
β-Carotene, μg/1000 kcal	1374.1 (1189.0)	1383.5 (1230.0)	1322.9 (937.2)	0.889
Vitamin C, mg/1000 kcal	66.4 (39.0)	66.6 (39.9)	65.2 (33.5)	0.845
Vitamin D, μg/1000 kcal	4.50 (3.53)	4.43 (3.48)	4.86 (3.79)	0.186

*BMI* body mass index, *SBP* systolic blood pressure, *DBP* diastolic blood pressure, *HbA1c* glycated hemoglobin, *HDL* high-density lipoprotein, *LDL* low-density lipoprotein, *SFA* saturated fatty acid, *MUFA* monounsaturated fatty acid, *PUFA* polyunsaturated fatty acid, *% energy* % total caloric intake.

Significant values are in bold.

### Associations between fatty acid intake and the prevalence of DR

**Figure 1:**
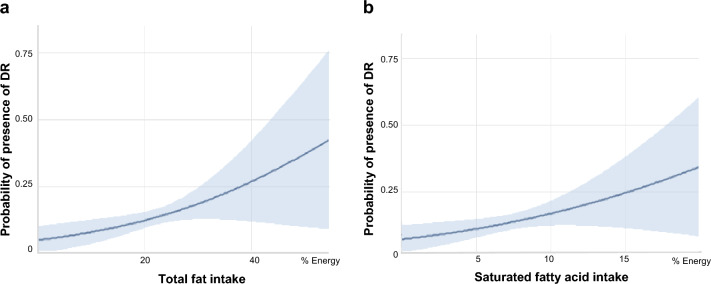
Estimated probabilities of presenting with diabetic retinopathy. Estimated probabilities of presenting with diabetic retinopathy by total fat intake (**a**) and saturated fatty acid intake (**b**). Logistic regression models were adjusted for age, sex, total energy, smoking status, alcohol intake, HbA1c, SBP, dyslipidemia, BMI, creatinine and intakes of α-tocopherol, β-carotene, vitamins C and D. DR, diabetic retinopathy.

The associations between specific types of dietary fatty acid intake and DR are shown in Table 2. After adjusting for age, sex, total energy intake, smoking status, alcohol intake, HbA1c, systolic blood pressure (SBP), dyslipidemia, BMI, creatinine, and vitamin intakes (α-tocopherol, β-carotene, vitamin C and vitamin D), the highest quartiles of the total fat and SFA intake were positively associated with the presence of DR compared with the lowest quartiles (odds ratio [OR], 2.61; 95% confidence interval [CI], 1.07–6.39; P for trend = 0.025; and OR, 2.40; 95% CI 1.12–5.17; P for trend = 0.013, respectively) (Table 2, Model 3) (Fig. 1). No significant associations were found between the prevalence of DR and the intakes of MUFA, PUFA, n3-PUFA, or n6-PUFA.

**Table 2:** The association between fatty acid intake and the presence of diabetic retinopathy.

Fatty acid intake	Quartile1	Quartile2	Quartile3	Quartile 4	*P* value for trend
Total fat					
Median (range) intake, % energy	14.37 (2.14, 17.47)	19.64 (17.49, 21.84)	23.74 (21.86, 26.30)	30.10 (26.36, 49.6)	
No. with DR/ at risk (%)	14/161	26/162	33/162	27/162	
Model 1, OR (95% CI)	Reference	**2.21 (1.09, 4.44)**	**3.16 (1.58, 6.32)**	**2.55 (1.23, 5.28)**	**0.009**
Model 2, OR (95%CI)	Reference	1.94 (0.91, 4.14)	**3.22 (1.52, 6.81)**	**2.25 (1.01, 4.98)**	**0.031**
Model 3, OR (95%CI)	Reference	2.02 (0.93, 4.40)	**3.53 (1.59, 7.84)**	**2.61 (1.07, 6.39)**	**0.025**
SFA					
Median (range) intake, % energy	4.26 (0.74, 5.40)	6.24 (5.42, 7.03)	7.93 (7.07, 8.80)	10.28 (8.81, 18.63)	
No. with DR/ at risk (%)	17/161	23/162	28/162	32/162	
Model 1, OR (95%CI)	Reference	1.52 (0.77, 3.00)	**1.99 (1.02, 3.87)**	**2.48 (1.27, 4.85)**	**0.006**
Model 2, OR (95%CI)	Reference	1.28 (0.61, 2.69)	1.91 (0.92, 3.95)	**2.35 (1.13, 4.86)**	**0.011**
Model 3, OR (95%CI)	Reference	1.24 (0.58, 2.65)	1.86 (0.87,3.99)	**2.40 (1.12, 5.17)**	**0.013**
MUFA					
Median (range) intake, % energy	5.75 (0.55, 7.03)	7.96 (7.04, 8.94)	9.84 (8.95,11.01)	13.02 (11.03, 24.45)	
No. with DR/ at risk (%)	18/161	24/162	31/162	27/162	
Model 1, OR (95%CI)	Reference	1.47 (0.76, 2.84)	**2.13 (1.11, 4.09)**	1.83 (0.93, 3.59)	**0.050**
Model 2, OR (95%CI)	Reference	1.36 (0.67, 2.77)	**2.03 (1.01, 4.07)**	1.59 (0.76, 3.32)	0.145
Model 3, OR (95%CI)	Reference	1.36 (0.66, 2.81)	2.06 (0.98, 4.36)	1.69 (0.73, 3.89)	0.157
PUFA					
Median (range) intake, % energy	3.60 (0.66, 4.42)	4.99 (4.42, 5.41)	5.94 (5.41, 6.47)	7.30 (6.50, 13.01)	
No. with DR/ at risk (%)	21/161	24/162	26/162	29/162	
Model 1, OR (95%CI)	Reference	1.38 (0.72, 2.62)	1.52 (0.80, 2.86)	1.61 (0.85, 3.05)	0.148
Model 2, OR (95%CI)	Reference	1.41 (0.70, 2.83)	1.25 (0.61, 2.54)	1.49 (0.73, 3.03)	0.369
Model 3, OR (95% CI)	Reference	1.45 (0.70, 2.99)	1.35 (0.60, 3.06)	1.65 (0.68, 4.01)	0.339
n3- PUFA					
Median (range) intake, % energy	0.57 (0.04, 0.74)	0.85 (0.74, 0.95)	1.05 (0.96, 1.23)	1.46 (1.23, 3.91)	
No. with DR/ at risk (%)	21/161	28/162	19/162	32/162	
Model 1, OR (95% CI)	Reference	1.42 (0.77, 2.63)	0.94 (0.48, 1.84)	1.78 (0.96, 3.31)	0.165
Model 2, OR (95% CI)	Reference	1.32 (0.68, 2.53)	0.82 (0.40, 1.68)	1.54 (0.79, 2.97)	0.404
Model 3, OR (95% CI)	Reference	1.29 (0.62, 2.65)	0.84 (0.35, 1.98)	1.57 (0.55, 4.50)	0.682
n6- PUFA					
Median (range) intake, % energy	2.94 (0.59, 3.57)	4.01 (3.57, 4.45)	4.81 (4.45, 5.23)	5.94 (5.24, 10.75)	
No. with DR/ at risk (%)	19/161	26/162	28/162	27/162	
Model 1, OR (95% CI)	Reference	1.81 (0.91, 3.61)	1.77 (0.89, 3.53)	1.90 (0.96, 3.76)	0.097
Model 2, OR (95% CI)	Reference	1.85 (0.92, 3.73)	1.75 (0.87, 3.53)	1.89 (0.95, 3.79)	0.115
Model 3, OR (95% CI)	Reference	1.76 (0.85, 3.65)	1.62 (0.73, 3.59)	1.75 (0.74, 4.14)	0.311

*DR* diabetic retinopathy, *OR* odds ratio, *SFA* saturated fatty acid, *MUFA* mono-unsaturated fatty acid, *PUFA* polyunsaturated fatty acid, *% energy* %energy of total calory intake.

Significant values are in bold.

Model 1: Adjusted for age, sex and total energy; Model 2: Adjusted for age, sex, total energy, smoking status, alcohol intake, HbA1c, SBP, dyslipidemia, BMI and Creatinine; Model 3: Adjusted for age, sex, total energy, smoking status, alcohol intake, HbA1c, SBP, dyslipidemia, BMI, Creatinine and intakes of α-tocopherol, β-carotene, vitamins C and D.

Further subgroup analyses of the association between SFA intake and DR were performed and stratified according to well-controlled (HbA1C < 7.0%) and poorly controlled (HbA1C ≥ 7.0%) diabetes. In patients with well controlled diabetes, an increased SFA intake tended to be associated with an increased odds of developing DR (model 3: p for trend = 0.095). Among the poorly controlled patients, the association was weaker, but a similar tendency was observed.

## Discussion

We analyzed a Japanese cohort of participants with diabetes and found that the intakes of total fat and SFA were positively associated with the presence of DR. Previous studies examining the association between SFA intake and DR have been limited, with inconsistent results^20,22^. A case–control study of 294 patients with type 2 Diabetes in Spain reported that the intake of trans fats and SFA was not associated with the prevalence of DR^22^. Meanwhile, a cross-sectional study of 379 patients with diabetes in Australia showed that SFA intake was associated with the prevalence of DR when glycemic control was good (HbA1C < 7.0%), whereas no association was evident when glycemic control was poor (HbA1C > 7.0%)^20^. The authors speculated that the harmful effects of hyperglycemia may counteract the influence of fatty acid intake in patients with poorly controlled diabetes. In the current study, patients with diabetes had relatively good glycemic control, with a mean HbA1c of 7.0%, which may partly explain the association between SFA intake and DR.

The role of SFA in health has been discussed extensively. The World Health Organization^28^ advocates that total fat should not exceed 30% of the total energy intake^29–31^, and that SFA intake should be less than 10%, with a shift in fat consumption away from SFA to unsaturated FA^31^ for the general population. Asians consume smaller amounts of SFA-containing foods than Western populations^17,18^.According to the US National Health and Nutrition Examination Survey, the mean intakes of total fat and SFA were 36.2% and 11.7% of the total energy intake, respectively, in American individuals aged 20 years or older^32^. Meanwhile, according to the National Health and Nutrition Survey in Japan, the mean intakes of total fat and SFA are 28.7% and 8.4% of the total energy intake in Japanese individuals aged 20 years or older^33^, respectively. In the present study, the mean intakes of total fat and SFA were 22.0% and 7.3% of the total energy intake, respectively, in patients with diabetes aged 40 years or older. Our results suggest that high fat intake may affect the prevalence of DR, even in patients with diabetes mellitus whose total fat intake is below the recommended levels.

No significant association was observed between PUFA intake and DR prevalence in the present study. Previous studies examining the association between the prevalence of DR and intake of PUFA and n-3 PUFA have shown inconsistent results^20–24^. A cross-sectional study in Australia reported that PUFA intake was inversely associated with the prevalence of DR when individuals with diabetes had good glycemic control^20^. The Prevención con Dieta Mediterránea (PREDIMED) trial, a randomized controlled study examining the effect of the Mediterranean diet on 3482 patients with type 2 diabetes, found that patients who consumed n-3 PUFA at or above the recommended dose for the prevention of cardiovascular disease (500 mg/day) had an approximately 50% lower risk of developing DR with visual impairment^21^. In the present study, the mean energy-adjusted n-3 PUFA intake was 2.3 g/day. We speculate that the lack of association between n-3 PUFA intake and the prevalence of DR in the present study may be partly due to the high consumption of n-3 PUFA over a potential threshold^9,34^. Further studies are required to clarify the association between PUFA intake and risk of DR.

Furthermore, in the current study, participants with DR exhibited lower serum triglyceride (TG) levels and a lower prevalence of dyslipidemia and were less likely to be current drinkers. There have been conflicting results regarding the association of serum TG levels and dyslipidemia with DR. Yao et al. reported that TG levels were inversely associated with DR, consistent with our study results^35^. However, a meta-analysis found no association of TG level or dyslipidemia with DR^1^. Several studies have reported a positive or negative association between alcohol intake and DR, but one meta-analysis found no such association^36^. Therefore, the results of this study do not necessarily contradict those of previous studies.

The strengths of this study include the use of standardized grading protocols to diagnose DR, as assessed by ophthalmologists, including retinal specialists, and the use of detailed questionnaires to collect lifestyle and medical history data. The validated the Food Frequency Questionnaire (FFQ) allowed the calculation of the intake of specific fatty acids. Our study has several limitations. First, the design of this study was cross-sectional, and we were unable to detect temporal information regarding associations. Second, we were unable to gather detailed information regarding diabetes status, including its duration, owing to the limitations of the data derived from a population-based cohort. Third, the correlation coefficients between fatty acid intake calculated from the FFQ and dietary records were lower in women than in men, which may have attenuated the association between fatty acid intake and DR in women. Fourth, in the current study, trans fatty acids and cellulose could not be analyzed due to a lack of data, and no studies to date have reported a certain relationship between trans fatty acid or cellulose intake and DR^37,38^. Finally, due to multicollinearity concerns, we did not include protein in the model, and no studies indicate a significant association between protein intake and DR after adjustment for confounders^38^. Nonetheless, it is crucial to recognize the potential confounding effect of nutrients. Therefore, future studies should investigate this possibility to gain further insights.

In summary, we found that total fat and SFA intakes were positively associated with the presence of DR. Our findings suggest that higher fat intake may affect the prevalence of DR, even in individuals whose total fat intake is generally much lower than that of Westerners and those who are more susceptible to developing diabetes. Although prospective longitudinal studies are needed to confirm this observation, these findings would enable us to understand the potential role of dietary fatty acid intake in the improved management of DR.

## Population and methods

### Study population

The JPHC-NEXT Eye Study is an ancillary study conducted as part of the JPHC-NEXT Study protocol^39^. Residents of Chikusei City, Japan, aged 40–74 years participated in this systemic and ophthalmological survey. The present study included 7090 individuals who participated in the survey between 2013 and 2015, of whom 5691 (80.3%) aged 40–74 years completed the FFQ. After excluding 14 participants because of missing or poor-quality fundus images, 647 participants with diabetes were included in the analysis.

This study was conducted in accordance with the Ethical Guidelines for Medical and Health Research Involving Human Subjects, Japan, and was approved by the Medical Ethics Committees of the School of Medicine, Keio University, Tokyo; the University of Tsukuba, Ibaraki; the University of Osaka, Osaka; and the National Cancer Center, Tokyo. Written informed consent was obtained from all the participants.

### Data and sample collection

Non-mydriatic fundus photographs of both eyes were taken using a 45° non-mydriatic fundus camera (Canon CR-1, Canon Inc., Tokyo, Japan) as part of eye screening. The images were centered on the optic disc and macula.

Blood samples were collected to measure serum glucose (fasting or nonfasting), HbA1c (%), TC (mmol/L), HDL-C (mmol/L), LDL-C (mmol/L), and TG (mmol/L). The non-fasting state was defined as fasting for < 8 h after the last meal. Diabetes was defined as the use of antidiabetic medication, or a fasting serum glucose ≥ 7.0 mmol/L or non-fasting serum glucose ≥ 11.1 mmol/L, or HbA1c ≥ 6.5% (National Glycohemoglobin Standardization Program)^40^. Dyslipidemia was defined as the use of lipid-lowering medication, or LDL-C ≥ 3.6 mmol/L or HDL-C < 1.0 mmol/L, or TG ≥ 1.7 mmol/L^41^. Blood pressure (BP) was measured twice on the right upper arm while the participant was seated. Mean values were used for the analysis. Hypertension was defined as the use of blood pressure medication, or a systolic BP ≥ 140 mm Hg or diastolic BP ≥ 90 mm Hg^42^. BMI was calculated as weight (kg) divided by height squared (m^2^).

### Grading of fundus photographs for DR

Any DR was defined as an Early Treatment Diabetic Retinopathy Study (ETDRS) levels ≥ 20 in either eye. The prevalence of DR was determined by two ophthalmologists who were blinded to the participants’ clinical data (T.K., H.T., E.Y., Y.K., K.M., or H.K.). In cases of disagreement, the diagnosis was made by a retinal specialist (Y.T. or N.O.).

### Dietary assessment

The dietary intake was evaluated using the long-form FFQ in the JPHC-NEXT study^43^. Briefly, the long-form FFQ comprises 172 food and beverage items and nine frequency categories, ranging from “almost never” to “seven or more times per day,” or to “10 or more glasses per day” for beverages. The questionnaire inquired about the usual consumption of the listed foods and beverages during the previous year. The nutrients, including fatty acids, in each food item were estimated using the fifth version of the Japan Food Table^44^. The nutrient intake was calculated by multiplying the frequency of consumption by the estimated intake for each food and summing across all items. Fatty acid intake was calculated as the percentage of total energy intake (nutrient density), and vitamin intakes were expressed as rates per 1000 kcal. They were divided into quantiles for further analysis. The validity of the FFQ for assessing fatty acid intake was confirmed using 12-day weighed food records (12d-WFR).^43^ The Spearman’s correlation coefficients for correlation between the energy-adjusted intake of fatty acids calculated from FFQ and dietary records ranged from 0.38 (for n-3 PUFA) to 0.55 (for MUFA) for men and from 0.21 (for MUFA) to 0.46 (for SFA) for women^43^, indicating moderate validity for fatty acids.

### Statistical analysis

Baseline characteristics were calculated for the overall sample and subgroups stratified according to the presence of DR (Table 1). Differences in basic characteristics between patients with and without DR were tested using the Wilcoxon rank-sum test for continuous variables and the chi-square test for categorical variables. Associations between fatty acid intake and DR prevalence were examined using multivariable logistic regression models and expressed as odds ratios (ORs) with 95% confidence intervals (CIs). In the first model, we adjusted for age, sex, and total energy intake, in the second model further for smoking status (current or non-current smokers) and alcohol intake (current or non-current drinkers), HbA1c, SBP, dyslipidemia, BMI, and creatinine, and the third model further for intakes of α-tocopherol, β-carotene, vitamins C and D. Statistical significance was set at P < 0.05. significant. All statistical analyses were performed using SAS for Windows, version 9.4 (SAS Institute, Inc., Cary, NC, USA).

## Data Availability

The data that support the findings of this study are available upon request from the corresponding author, MS, or KY. The data are not publicly available because they contain information that can compromise the privacy of the research participants.
